# Novel Silica Hybrid Xerogels Prepared by Co-Condensation of TEOS and ClPhTEOS: A Chemical and Morphological Study

**DOI:** 10.3390/gels8100677

**Published:** 2022-10-20

**Authors:** Guillermo Cruz-Quesada, Maialen Espinal-Viguri, María Victoria López-Ramón, Julián J. Garrido

**Affiliations:** 1 Institute for Advanced Materials and Mathematics (INAMAT2), Department of Science, Public University of Navarre (UPNA), Campus Arrosadia, 31006 Pamplona, Spain; 2 Department of Inorganic and Organic Chemistry, Faculty of Experimental Sciences, University of Jaen, 23071 Jaen, Spain

**Keywords:** xerogels, hybrid materials, TEOS, chlorophenyltriethoxysilane, chemical-textural properties, ORMOSILs, ordered structures, silica species

## Abstract

The search for new materials with improved properties for advanced applications is, nowadays, one of the most relevant and booming fields for scientists due to the environmental and technological needs of our society. Within this demand, hybrid siliceous materials, made out of organic and inorganic species (ORMOSILs), have emerged as an alternative with endless chemical and textural possibilities by incorporating in their structure the properties of inorganic compounds (i.e., mechanical, thermal, and structural stability) in synergy with those of organic compounds (functionality and flexibility), and thus, bestowing the material with unique properties, which allow access to multiple applications. In this work, synthesis using the sol-gel method of a series of new hybrid materials prepared by the co-condensation of tetraethoxysilane (TEOS) and 4-chlorophenyltriethoxysilane (ClPhTEOS) in different molar ratios is described. The aim of the study is not only the preparation of new materials but also their characterization by means of different techniques (FT-IR, ^29^Si NMR, X-ray Diffraction, and N_2_/CO_2_ adsorption, among others) to obtain information on their chemical behavior and porous structure. Understanding how the chemical and textural properties of these materials are modulated with respect to the molar percentage of organic precursor will help to envisage their possible applications: From the most conventional such as catalysis, adsorption, or separation, to the most advanced in nanotechnology such as microelectronics, photoluminescence, non-linear optics, or sensorics.

## 1. Introduction

**Figure 1:**
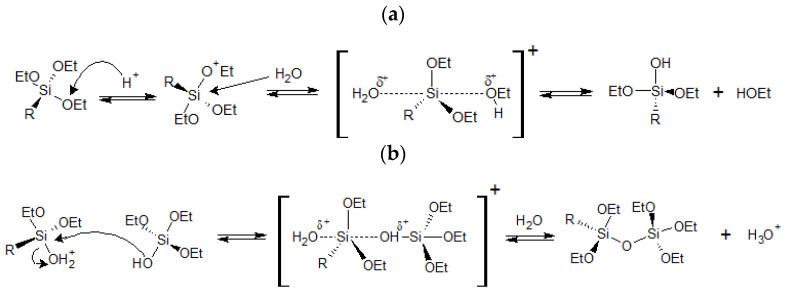
Mechanism in acidic media of (**a**) the first hydrolysis reaction of tetraethoxysilane (R = OEt) or triethoxysilane (R = alkyl or aryl), and (**b**) the co-condensation reaction of tetraethoxysilane with a triethoxysilane (R = alkyl or aryl).

Silica hybrid gels are among the so-called organic modified silica (ORMOSILs). Nowadays, these materials are being used for multiple applications such as the adsorption of metals in water [1,2,3], catalyst support [4,5,6], support for luminescent compounds [7,8,9], anti-corrosive or fungicide coatings [10,11,12,13], and biomedicine [14,15,16,17,18,19]. The key behind the tunability of the properties of these materials is the synergetic effect that generates the coexistence, at a nanometric scale, of an inorganic skeleton with flexible and functional organic constituents [20,21]. The hybrid gels are prepared by the sol-gel method, a very versatile synthetic technique that allows different approaches: (i) Anchoring of organic molecules in the porous of the already-formed solid gel [1,2,7,8,9], (ii) grafting of organic moieties on the surface of a silica material [4,22,23], (iii) co-condensation of an organosilane (SiR_x_(OR’)_4−x_), with a carbon precursor, to form Si-O-C xerogels [16,18,24,25], or finally, (iv) classic co-condensation of a tetraalkoxysilane (Si(OR)_4_), usually tetraethoxysilane (TEOS), with one or more organosilanes [8,9,10]. In the latter approach, to obtain materials with the desired properties, it is of paramount importance to control the precursors’ hydrolysis and condensation reactions (Figure 1), due to their susceptibility to the media conditions (pH, solvents, use or not of a catalyst, proportions of H_2_O/precursor or organosilane/tetra alkoxysilane) [26,27,28,29,30].

The influence of these parameters in the synthesis of silica xerogels has been studied by our research group in recent years [31,32,33,34], with special emphasis on the nature and molar proportion of organosilanes in hybrid xerogels [35,36,37]. By means of different techniques, such as mass spectroscopy, inelastic neutron scattering, and infrared spectra deconvolution, it was confirmed that ordered domains of polyhedral oligomeric silsesquioxanes (POSS) are formed within the silica matrix of the hybrid materials, due to the blocking of the condensation direction in the organic precursor RTEOS (RTEOS = alkyl/chloroalkyl triethoxysilanes) and the intermolecular forces (e.g., hydrophobic or electrostatic interactions) exerted by the organic moieties [38,39,40]. In fact, ordered domains were detected at lower molar percentages of organic precursor when chloroalkyltriethoxysilanes (ClRTEOS) are used instead of their analogous alkoxysilanes [37,40]. The knowledge acquired in those studies has been used to prepare optical fiber sensors (OFS) by dip-coating, using selected gels with specific properties. These sensors have a labile and specific interaction with different adsorbates, such as vapor of water and volatile organic compounds (COVs), allowing their detection and monitoring [41,42,43].

Following this line of research, the aim of the present work is to study the morphological, textural, and chemical properties of silica hybrid xerogels containing a chlorophenyl moiety, and to evaluate their use as coatings for OFS in the future. For this purpose, a new series of transparent hybrid xerogels were prepared by co-condensation, in acidic media, of TEOS with (*p*-chlorophenyl) triethoxysilane (ClPhTEOS) at different molar percentages. The resulting materials were fully characterized by ^29^Si NMR (Nuclear Magnetic Resonance), XRD (X-ray Diffraction), FTIR (Fourier-Transform Infrared Spectroscopy), Helium Pycnometry, FE-SEM (Field-Emission Scanning Electron Microscopy), HR-TEM (High-Resolution Transmission Spectroscopy), and the adsorption of N_2_ (−196 °C) and CO_2_ (0 °C). In addition, a modified and adjusted deconvolution method of the FT-IR spectra was used to acquire semi-quantitative information about the proportion of (SiO)_4_ and (SiO)_6_ rings [40,44], which are related to ordered structures and amorphous silica, respectively. Finally, the results were compared with those of the previously studied xerogels to remark on the influence of the chlorophenyl moiety on the material’s properties.

## 2. Results and Discussion

### 2.1. FT-IR

**Figure 2:**
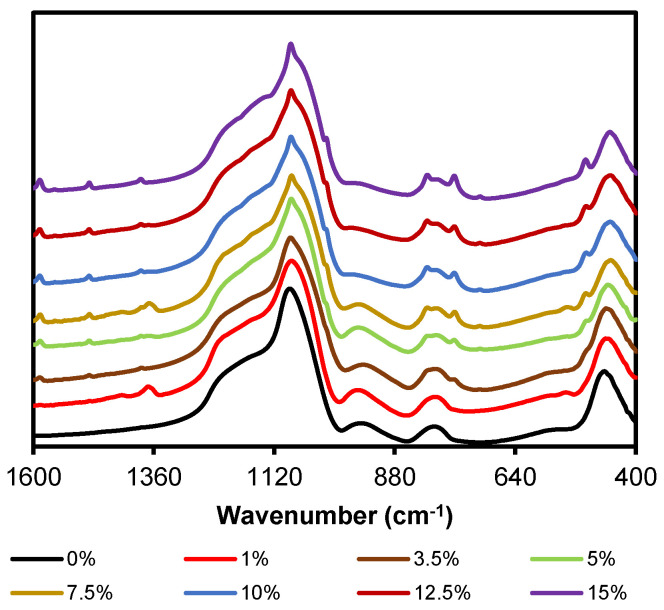
FT-IR spectra (range 1600–400 cm^−1^) of the reference (100%TEOS) and the hybrid materials at different molar percentages of organic precursor (ClPhTEOS).

Figure 2 depicts the FT-IR spectra in the range of 1600–400 cm^−1^ of the hybrid xerogels synthesized at different molar percentages of organic precursor. The spectra in the range of 4000–2750 cm^−1^ are displayed in Figure S1 of the Supplementary Material.

In Figure 2, the characteristic bands of the amorphous silica matrix can be observed: (i) Rocking of O–Si–O at 455 cm^−1^ (ρ O–Si–O), (ii) symmetric Si–O–Si stretching vibration at 800 cm^−1^ (ν_s_ Si–O–Si), (iii) Si-O bond stretching vibration of the silanols on the surface at 955 cm^−1^ (ν_s_ Si–OH), (iv) asymmetric Si–O–Si stretching vibration at 1090 cm^−1^ (ν_as_ Si–O–Si), and (v) a wide and intense shoulder from 1350 to 1120 cm^−1^ associated with various vibrational modes of the Si–O–Si [45]. Additionally, a slight shoulder at 550 cm^−1^ can be observed, which is associated with the presence of 4-member siloxane rings, (SiO)_4_ [46]. In the spectra, as the molar percentage of organic precursor gradually increases, a new incipient shoulder becomes more evident at 1140 and 1155 cm^−1^, and the band at 1080 cm^−1^ narrows and stands out, similar to an emerging peak. These changes could indicate that the organic precursor is favoring the formation, within the silica matrix, of new structures with vibration modes (ν_as_ Si–O–Si) at different specific frequencies [40]. In the spectral region of 400–2750 cm^−1^ (Figure S1 of the Supplementary Material), it is possible to observe the stretching bands of superficial silanols (ν Si–OH a 3450 cm^−1^) and those resulting from the interaction of these groups through hydrogen bonds (ν Si–OH–H at 3660 cm^−1^) [45].

**Figure 3:**
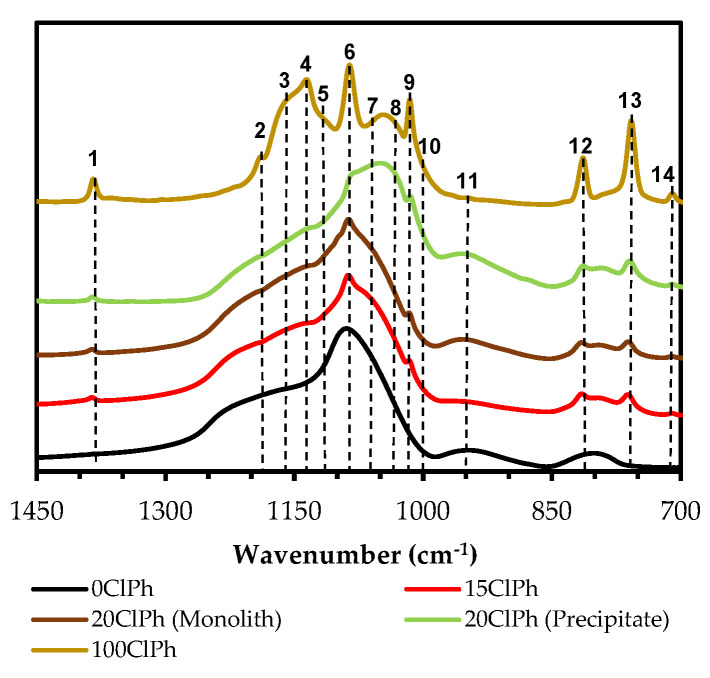
FT-IR spectra (range 1400–900 cm^−1^) of the reference, 15ClPh, and both phases of 20ClPh and 100ClPh.

The presence of the chlorophenyl moiety in the hybrid materials can be confirmed by a set of bands observed in the spectra: (i) The stretching bands of the hydrogens of the benzene ring in the range of 3090–3010 cm^−1^ (ν (C-H), Figure S1), (ii) three C=C bond stretching bands in the spectral range of 1450–1000 cm^−1^ (1380, 1085 and 1015 cm^−1^), and finally, (iii) three bands corresponding to the deformation vibrations of the C-H bonds in the aromatic rings (at 815, 760, and 500 cm^−1^), and a band due to the stretching vibration of the C–Cl bond (at 710 cm^−1^) [43,47,48]. It should be noted that the spectrum of the xerogel 20ClPh is not shown in Figure 2 because a heterogeneous monolith with two well-differentiated phases was obtained: A non-colored transparent phase above (monolith) and an opaque phase below (precipitate), whose spectra turned out to be very different (Figure S2c in the Supplementary Material). This difference might be because the organic precursor favors the formation of structures with lower solubility in the reaction media, and thus precipitates. To test this hypothesis and obtain information on those structures, a material using only ClPhTEOS was synthesized, resulting in a white, soft, and extremely hydrophobic solid (100ClPh) (Figure S2d in the Supplementary Material). Figure 3 depicts the FT-IR spectra of the reference, 15ClPh, 20ClPh (monolith and precipitate), and 100ClPh, while Table 1 displays the list of bands observed in the spectrum of 100ClPh, the vibrations and structures assigned to those bands, and the literature consulted for the assignment.

**Figure 4:**
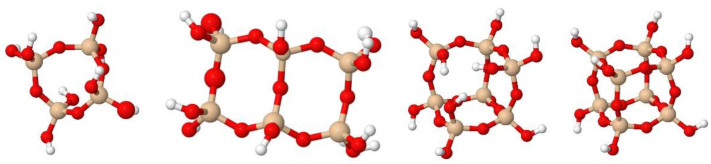
From left to right: 4-fold ring, short ladder (SLd), open-cage (T_7_), and close-cage (T_8_).

The bands due to the vibrations of ν_as_ (Si–O–Si) in the spectral range of 1400–1000 cm^−1^ (Table 1) correspond to simple structures (linear siloxane chains and (SiO)_4_ or (SiO)_6_ rings) and more compact and complex structures (i.e., polyhedral oligomers of silsesquioxanes, POSS). Oligomers known as open or closed cages (T_7_ and T_8_, respectively) and short ladders (SLd) are among the best known POSS, which are formed by the fusion of two or more four-membered rings ((SiO)_4_) (Figure 4) [52,53,54,55,56].

**Figure 5:**
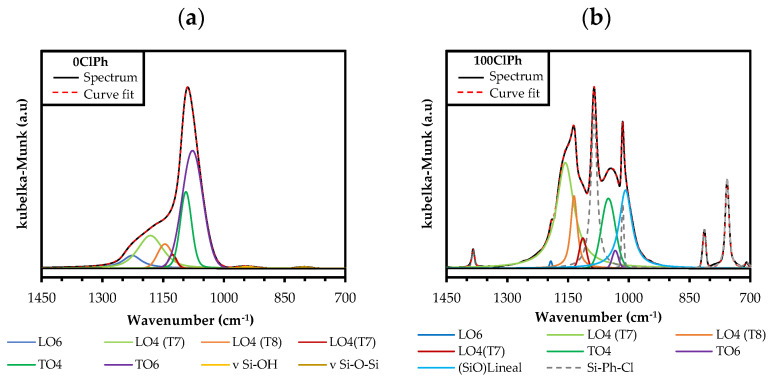
Experimental and calculated spectra of (**a**) reference and (**b**) 100ClPh.

The formation of the rings that constitute these structures ((SiO)_4_) is thermodynamically favored in the oligomerization of siloxanes in acidic media [45,57], in contrast to the six-membered rings ((SiO)_6_), kinetically favored and typical of amorphous materials [39,58]. To study how the precursor affects the formation of ordered structures, it is necessary to know the proportion of (SiO)_4_ in the silica matrix. For this purpose, the deconvolution of the FT-IR spectra in the range of 1300–980 cm^−1^ was performed using the non-linear least-squares method, obtaining the Gaussian–Lorentzian components. The different distances and degrees of torsion of Si–O–Si bonds in (SiO)_4_ and (SiO)_6_ rings allow us to distinguish bands belonging to the optical modes of vibration; two in transverse mode between 1100 and 1000 cm^−1^ (TO_4_ and TO_6_) and two in longitudinal mode between 1250 and 1100 cm^−1^ (LO_4_ and LO_6_) [40,44,59,60]. In this work, a modification of this method has been carried out, consisting of the adjustment of additional bands (Table 1) corresponding to (i) C=C and C-H vibrations of the chlorophenyl (1450–700 cm^−1^); (ii) vibrations of the siloxane groups and Si-O bonds, characteristic of amorphous silica (950 and 800 cm^−1^, respectively); (iii) vibrations of the (SiO)_4_ rings that make up the POSS: ν_ring-s_ and ν_ring-as_ for the “open” species (T_7_ and short ladders), and only ν_ring-as_ for the “closed” species (T_8_), due to the ν_ring-s_ vibration mode being forbidden [49]; and (iv) ν_as_ (Si–O–Si) vibration of linear siloxanes. As an example, the calculated spectra and the bands generated for the reference xerogel and 100ClPh are displayed in Figure 5. The calculated bands and the fit of 5ClPh, 10ClPh, 15ClPh, and the two phases of 20ClPh are depicted in Figure S3 of the Supplementary Material.

The percentage area of each component and the residual value (difference between the real spectrum and the fit) are exhibited in Table S1 of the Supplementary Material. The percentage of four-membered and six-membered rings was determined by applying the following equations to the Gaussian–Lorentzian bell areas of the TO and LO components:

(1) $${\left(\mathrm{SiO}\right)}_{6},\;\%=\frac{A\left({\mathrm{LO}}_{6}\right)+A\left({\mathrm{TO}}_{6}\right)}{A\left({\mathrm{LO}}_{4}\right)+A\left({\mathrm{TO}}_{4}\right)+A\left({\mathrm{LO}}_{6}\right)+A\left({\mathrm{TO}}_{6}\right)}\times 100$$

(2) $${\left(\mathrm{SiO}\right)}_{4},\;\%=\frac{A\left({\mathrm{LO}}_{4}\right)+A\left({\mathrm{TO}}_{4}\right)}{A\left({\mathrm{LO}}_{4}\right)+A\left({\mathrm{TO}}_{4}\right)+A\left({\mathrm{LO}}_{6}\right)+A\left({\mathrm{TO}}_{6}\right)}\times 100$$

where A(LO)_6_ is the area of the band at 1190 cm^−1^, (TO)_6_ is the area of the band at 1030 cm^−1^, A(LO)_4_ is the sum of the areas of the three LO_4_ bands, 1160, 1135, and 1120 cm^−1^, and A(TO)_4_ is the area of the band at 1050 cm^−1^. Table 2 displays the proportion of rings calculated by applying Equations (1) and (2). It can be observed that increasing the molar percentage of ClPhTEOS in the xerogels favors the formation of (SiO)_4_ rings (from 46.54% in the reference to 97.35% in 100ClPh).

Another noteworthy observation is the great difference in the percentage of these rings between both phases of 20ClPh: The monolithic phase has a percentage of rings similar to that of the reference 0ClPh, and the phase that precipitates is similar to 100ClPh. This might be explained by taking into account that an increase in ClPhTEOS favors the formation of POSS, which are mainly formed by four-membered rings, and, as their abundance increases, there is a critical point at which these species exceed the molar solubility in the reaction media and segregate as a precipitate [61].

### 2.2. ^29^Si Nuclear Magnetic Resonance (NMR)

**Figure 6:**
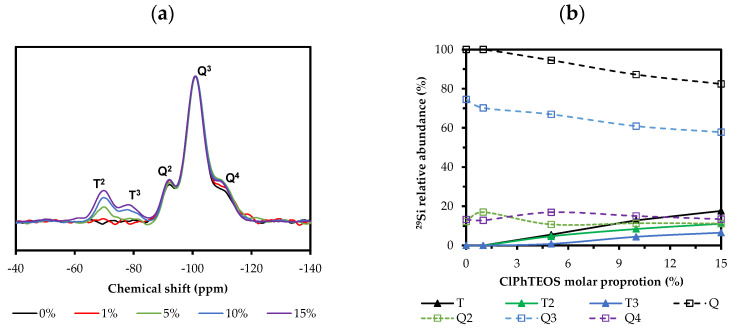
(**a**) Normalized ^29^Si NMR spectra of the hybrid xerogels. (**b**) Variation of the relative abundance of the condensed species with respect to the percentage of ClPhTEOS.

^29^Si NMR spectra of the hybrid materials were obtained to determine the relationship between the molar percentage of the precursor and the relative abundance of silicon species in the xerogels. Figure 6a depicts the spectra of the hybrid materials normalized with respect to the signal of the dominant species, Q^3^, the most intense in all cases.

The signals associated with the less condensed species (Q^1^ and T^1^) in Figure 6a are not observed. The dominant species corresponding to the hybrid precursor is the semi-condensed T^2^, whose intensity is greater than that of the more condensed T^3^ in all the materials. In addition, Figure 6b displays both the evolution of the relative abundance of Q (Q^2^ + Q^3^ + Q^4^) and T (T^2^ + T^3^) species with respect to the molar percentage of ClPhTEOS, as well as that of each species. For example, the Q^2^ relative abundance increases slightly to stabilize at 11% and Q^4^ increases up to 5% ClPhTEOS and then decreases at higher molar percentages of organic precursor. Table 3 exhibits the chemical shifts of each ^29^Si species in the spectra and the integrals of the T species.

There is no significant displacement of the chemical shifts with the increase in ClPhTEOS, indicating that the environment of the silicon atoms does not change substantially. The chemical shifts of the T signals (organic precursor) are less negative than those of the Q signals (TEOS) because the chlorophenyl moiety removes less electronic charge from the silicon atom than oxygen, favoring the Shielding Effect [62,63]. Additionally, a higher positive charge density in the silicon atom favors nucleophilic attacks and therefore condensation [64,65]; however, the more abundant species is the least condensed and not T^3^, indicating that the inductive effect exerted by the chlorine atom of the chlorophenyl moiety is weaker than its steric effect, preventing total condensation in the materials. The increase in T^3^ species is related to the presence of POSS in the material, since the silicon atoms that form (SiO)_4_ rings are mainly condensed species T^3^, Q^3^, or Q^4^ (less condensed structures T_7_ and SLd also contain T_2_ and Q_2_) [49]. In fact, the shifts of T^3^ species (Table 3) are closer to those observed in T^8^ structures (−77 ppm) than to those of the aliphatic R-Si-O_1_._5_ species (−66 to −67 ppm) [66,67]. Finally, it is worth mentioning that 100ClPh material only contains T units and, as has been verified in the analysis of its FT-IR spectra, it is composed almost exclusively of (SiO)_4_ rings. 

### 2.3. X-ray Diffraction (XRD)

**Figure 7:**
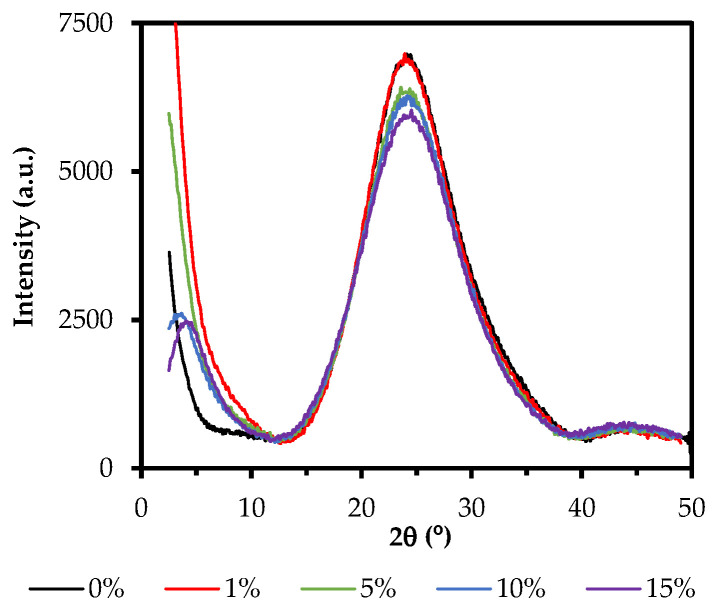
XRD diffractograms of the hybrid xerogels (reference, 1ClPh, 5ClPh, 10ClPh, and 15ClPh).

Figure 7 depicts the X-ray diffraction patterns of the hybrid materials synthesized at different molar percentages of organic precursor.

All the diffractograms showed a broad diffraction maximum at 2θ~24°, characteristic of the amorphous silica and associated with the distance between the silicon atoms linked by siloxane bridges [68]. This maximum slightly decreases with the increase in the molar percentage of the organic precursor. Interestingly, another maximum can be observed at 2θ < 10° when the molar percentage of the precursor is increased (10ClPh and 15ClPh). This new diffraction maximum is associated, in the literature, with the presence of ordered domains composed of POSS in the form of cages (T_7_ or T_8_) or short ladders within the amorphous matrix of the material [60]. The emergence of this maximum as the one at 24° decreases is consistent with the greater local structuration of the materials as we increase the molar percentage of the organic precursor. This behavior was already seen in previously studied ClRTEOS xerogels (R = M, methyl; E, ethyl, or P, propyl), where it was found that the maximum at 2θ < 10° appeared at lower molar percentages than their analogous RTEOS. In these chlorinated series, the minimum molar percentages containing the new maximum were 30, 1, and 10% for ClMTEOS, ClETEOS, and ClPTEOS, respectively [37]. The ClPhTEOS series not only share this minimum percentage with the ClPTEOS but also have more T^2^ than T^3^ species, which could indicate that, although the chlorine atom in the precursors favors the condensation and the subsequent formation of POSS, the steric effect of the bulkier organic moieties (both propyl and phenyl) could generate less condensed POSS, such as T_7_ or SLd. In all the patterns, another low-intensity maximum is observed around 45°, which is associated, according to Bragg’s law (*n* = 2 in *n*λ = 2*d* sin *θ*), with a replica of the maximum at 2θ~24°, indicating long-range order in the materials. Table 4 displays the angles, intensities, and distances calculated from Bragg’s law for each maximum in the diffractograms of Figure 5.

In 1ClPh and 5ClPh materials, the Si–O–Si bond elongates proportionally to the molar percentage of the precursor (maximum shifts to smaller angles), consistent with what was observed in the ^29^Si NMR spectra, that is, the increase in T species (from the organic precursor) causes a decrease in the average positive charge density of the silicon atoms, and therefore, the siloxane bonds are less polarized. However, as the molar percentage of the organic precursor increases in 10ClPh and 15ClPh, the bond becomes shorter (the maximum shifts to higher angles). This effect, inverse to the one discussed above, is related to the appearance of a diffraction maximum at 2θ < 10° in 10ClPh and 15ClPh, since this maximum is associated with ordered POSS-type structures, where the siloxane bridges (Si–O–Si) that make up the (SiO)_4_ rings are more compact than those of the amorphous silica (formed mainly by (SiO)_6_) [40], thus explaining the decrease in Si–O–Si distances associated with the maximum at 2θ~24°. The calculated distances for the additional maximum at 2θ < 10° (displayed in Table 4) are similar to those associated with the organic moiety in the cage-like structures (1–3 nm) [39,54], and to that of the interplane between short ladders [56,69]. Additionally, the X-ray diffractogram of 100ClPh is depicted in Figure S4 (Supplementary Material). In the diffractogram, a sharp and intense diffraction maximum at 2θ = 6.84° (1.3 nm) can be observed, which confirms that the 20ClPh precipitate contains a large amount of POSS. In a recent study, Nowacka et al. reported an interplane distance of 1.24 nm for ladder-like phenylsilsesquioxane oligomers, suggesting that these are the species formed in 100ClPh [60].

### 2.4. Helium Pycnometry

**Figure 8:**
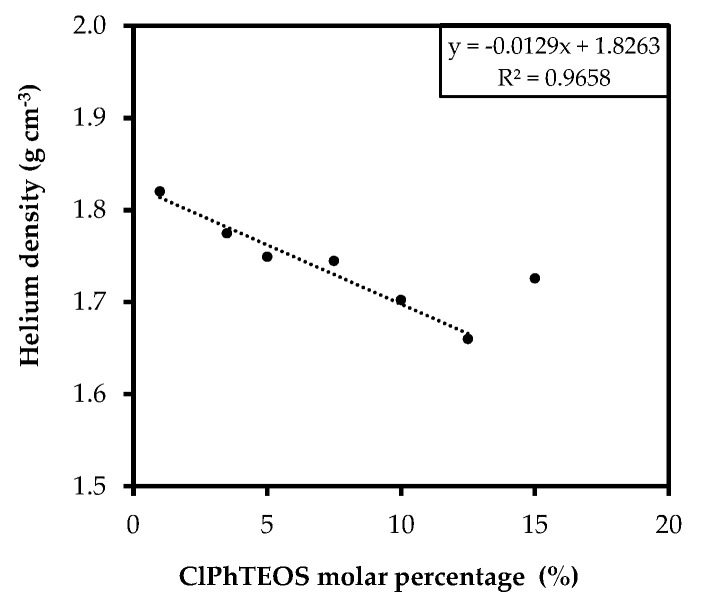
Skeletal density of the hybrid materials with respect to the percentage of ClPhTEOS.

Helium pycnometry reveals the skeletal density of the synthesized xerogels. Figure 8 depicts the variation in the skeletal density as a function of the molar percentage of ClPhTEOS.

A decrease in the skeletal density with respect to ClPhTEOS is observed, since the organic precursor blocks one of the hydrolysis and condensation positions, thus reducing the degree of cross-linking with respect to the reference material (100% tetraethoxysilane, TEOS). The skeletal density of 15ClPh is greater than expected, which indicates that the limit of precursor that the system can assimilate has been reached. This new series has lower values of skeletal density than the previously studied ClRTEOS series (R = methyl, M; ethyl, E; propyl, P). For example, for 10ClPh, the skeletal density is 1.68 in contrast to 1.91, 1.85, and 1.80 g cm^−1^ for ClMTEOS, ClPTEOS, and ClETEOS, respectively [37]. This result is consistent with the information deduced from the ^29^Si NMR spectra and the XRD diffractograms (ClPhTEOS favors the less condensed POSS, T_7_ and SLd).

### 2.5. N_2_ and CO_2_ Adsorption Isotherms

N_2_ and CO_2_ molecules have a similar size; however, the temperature at which the adsorption takes place is very different, being −196 °C for N_2_ isotherms and 0 °C for CO_2_ isotherms. If the pores are very thin, N_2_ molecules cannot access them due to kinetic restrictions; however, CO_2_ molecules can. On the other hand, the high saturation pressure of CO_2_ vapor (3.5 MPa) allows the finer microporosity to be explored in detail, which is covered at very low relative pressures. This fact makes the data provided by both isotherms complementary and makes it possible to differentiate the micropores of less than 0.7 nm and even the finest mesopores. If there are no kinetic constraints, N_2_ adsorption provides the volume of pores of less than 50 nm, CO_2_ adsorption of those sized less than 0.7 nm, and the difference of these values would provide the microporosity between 2 and 0.7 nm. This divergence can be explained by the different adsorption mechanisms taking place in both microporous intervals; in the so-called “primary micropore filling”, the ultramicropores are accessed at a very low relative pressure (*p*/*p*_0_ = 0.03) and the adsorbent–adsorbate interactions predominate over those of the adsorbate–adsorbate, whereas, in wider micropores, the adsorbate–adsorbate interactions predominate, favoring a cooperative process of adsorption [70,71].

**Figure 9:**
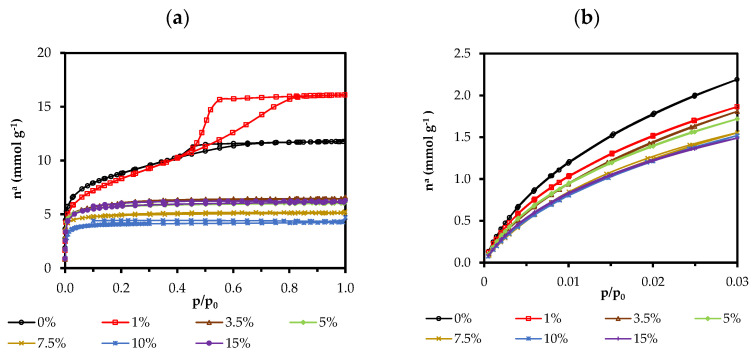
Isotherms of the hybrid xerogels: (**a**) N_2_ (−196 °C), and (**b**) CO_2_ (0 °C).

The N_2_ adsorption isotherms (at −196 °C) and the CO_2_ isotherms (at 0 °C) of the hybrid xerogels are shown in Figure 9. The isotherm of the reference material has an open knee, a sign of a wide micropore size distribution, characteristic of type I(b) isotherms. Moreover, its slope is pronounced in the adsorption, and it presents a hysteresis loop in the desorption (H2(a)), which is characteristic of a type IV isotherm typical of mesoporous materials. Therefore, the reference material can be considered micro-mesoporous with a type I(b)-IV(a) mixed isotherm [71]. Except for 1ClPh and 15ClPh materials, the higher the percentage of an organic precursor the smaller the pore volume, obtaining a type I(a) isotherm in all cases, typical of microporous materials with a narrow pore distribution. The isotherm of 1ClPh is a type IV(a) with an H1 hysteresis loop, indicating that, in this case, the organic precursor increases the total volume of pores and mesopores with respect to the reference, an effect that was also observed in analogous hybrid materials previously reported, which is associated with a change in the morphology of the pores, from cone-shaped to inkwell-shaped pores [27,37]. The 15ClPh isotherm is type I(a) but, surprisingly, it adsorbs more N_2_ than 10ClPh, 7.5ClPh, and ClPh5. This fact is consistent with the value obtained for its skeletal density (Figure 8), which suggests that in this material, the limit of the organic precursor that the xerogel accepts is practically reached and is therefore heterogeneously distributed in the silicon matrix. Table 5 exhibits the textural parameters obtained from the adsorption isotherms.

**Figure 10:**
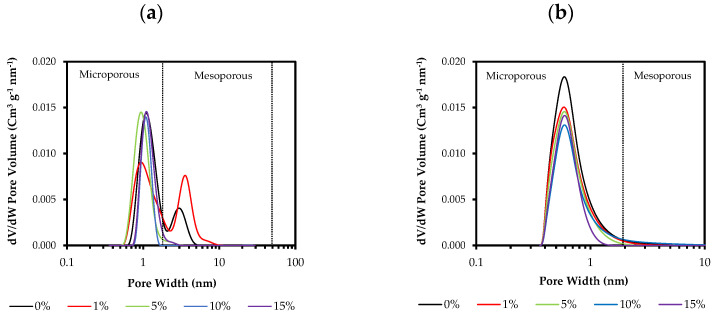
Pore size distribution of the materials calculated from (**a**) N_2_ isotherms and (**b**) CO_2_ isotherms.

A decrease in the specific surface area (a_BET_) with the increase in the molar percentage of ClPhTEOS is observed, except for 15ClPh, which has a larger area than expected. Additionally, the table displays the volume of micropores obtained from the N_2_ adsorption data (V_micro_(N_2_)), and of the narrowest micropores (V_micro_(CO_2_), where φ < 0.7 nm), determined by applying the Dubinin–Raduskevich equation to the CO_2_ adsorption data. Both volumes decrease with an increase in the molar percentage of ClPhTEOS. The average pore size determined by the Barrett–Joyner–Halenda method (BJH APS) indicates that their mesoporosity narrows with the molar percentage of ClPhTEOS until microporous materials are obtained. In comparison with the ClRTEOS materials with the same percentage of the organic precursor, 10ClPh has a higher surface area and V_micro_(N_2_) than those of ClPTEOS and ClETEOS, but lower than that of ClMTEOS [37]. This implies, once again, that these materials are less condensed due to the predominance of T^2^ over T^3^ species and the bulky nature of the chlorophenyl group. Figure 10 depicts the pore size distribution by applying DFT calculations to the N_2_ and CO_2_ isotherm data.

All the materials are microporous, with an internal width close to 1 nm (Figure 10). The distribution shows that the materials do not present a significant volume of mesopores, except for the reference and 1ClPh.

### 2.6. Microscopy

#### 2.6.1. Field-Emission Scanning Electron Microscopy (FE-SEM)

**Figure 11:**
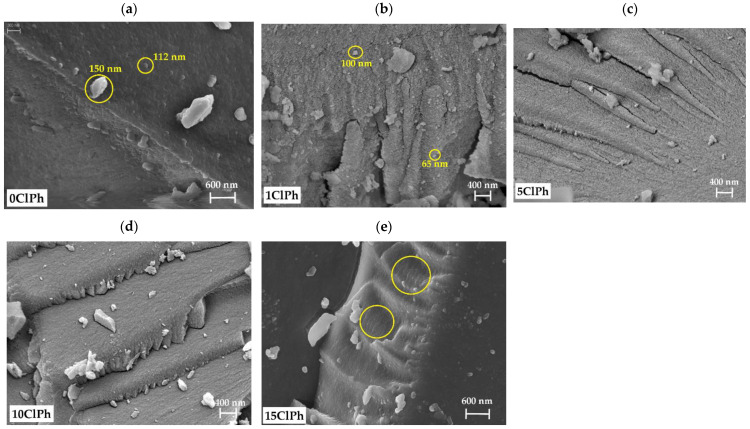
FE-SEM micrographs of: (**a**) 0ClPh, (**b**) 1ClPh, (**c**) 5ClPh, (**d**) 10ClPh, and (**e**) 15ClPh.

To determine the influence of the precursor on the texture, micrographs of the materials were acquired using scanning electron spectroscopy (SEM). The micrographs of the whole series are displayed in Figure 11.

The micrograph of the reference material (Figure 11a) exhibits a rough morphology composed of small intersecting globular particles in a chainmail-like surface (110–150 nm). The space between the particles corresponds to the narrow mesoporosity of this material, consistent with the N_2_ adsorption isotherm. Figure 11b shows how the use of only 1% of the organic precursor in the synthesis of 1ClPh results in a remarkable change: The surface is rougher, and the constituent particles are smaller (30–100 nm), justifying why the isotherm of this material reflects a greater specific surface area and mesoporosity than the reference material (0ClPh). In Figure 11c,d, respectively, ClPh5 and ClPh10 display a progressive smoothing of the surface with the increase in the organic precursor, which is consistent with the loss of mesoporosity and pore volume reduction deducted from their isotherms. A stratification of the surface can also be observed in overlapped compacted sheets to give mainly microporous materials. In ClPh15 (Figure 11e), the layers of compacted sheets, which are related to the stacking of the ladder POSS [56], can be better appreciated.

#### 2.6.2. High Resolution-Transmission Electron Microscopy (HR-TEM)

**Figure 12:**
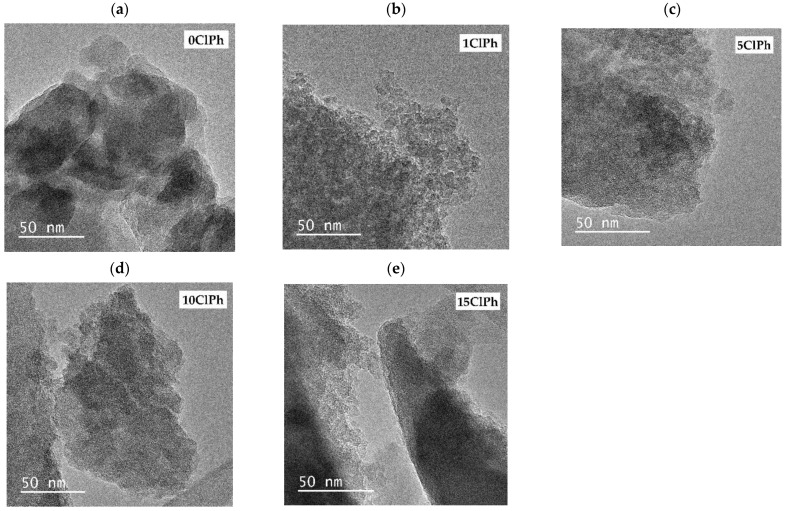
HR-TEM micrographs of (**a**) 0ClPh, (**b**) 1ClPh, (**c**) 5ClPh, (**d**) 10ClPh, and (**e**) 15ClPh.

Figure 12 displays TEM micrographs of the hybrid materials.

The micrograph of the reference material (Figure 12a) exhibits a morphology formed by an agglomerate of large particles of several tens of nm, which leave holes that are related to the mesoporosity of the material. In the following micrograph (Figure 12b, 1ClPh), particles of smaller size are interconnected, but they are still identifiable, unlike in the micrographs of the materials with a higher percentage of ClPhTEOS. To determine the degree of precursor distribution in the material, a mapping of the weight percentage of chlorine in the materials was carried out by X-ray energy dispersion. Table 6 displays the experimental and theoretical weight percentage of chlorine obtained for each material.

The weight percentages of chlorine in Table 6 are the average of the values recorded in different particles of the sample and reflect that the actual content of chlorine in the materials is lower than the theoretical. However, most of the chlorophenyl precursor is found as part of 1ClPh, 5ClPh, and 10ClPh materials, in contrast to 15ClPh, where approximately half of the amount of precursor used for the synthesis is present in the material. This is consistent with what was observed in the rest of the characterization techniques, since the results obtained are characteristic of a material with a lower molar percentage of the organic precursor than the actual amount used for its preparation. 

## 3. Conclusions

A new series of hybrid xerogels have been prepared via the co-condensation of TEOS and ClPhTEOS as the organic precursor at different molar percentages. From the FT-IR spectra of the materials and by applying a deconvolution method, the percentages of (SiO)_6_ and (SiO)_4_ rings in the silica matrix of the materials were determined, the former associated with amorphous silica and the latter with ordered structures such as polysilsesquioxanes (POSS). The study determined that increasing the molar percentage of the organic precursor leads to an increase in the proportion of four-membered rings, (SiO)_4_. The presence of POSS is consistent with the XRD diffractograms, where an additional maximum at 2θ < 10° associated with ordered domains is observed for the materials with the highest ClPhTEOS content. The ^29^Si NMR spectra indicate that the most abundant silicon species from the organic precursor (T) are the semi-condensed (T^2^), likely due to the steric and electronic effect exerted by the chlorophenyl groups during the crosslinking of the colloids. Regarding the textural properties, it has been determined that an increase in the organic precursor in the materials results in a loss of mesoporosity and specific surface area, and a narrower pore size distribution. This is closely related to the smoothing of the material’s surface and the decrease in particle size observed in the FE-SEM and HR-TEM when increasing the molar percentage of ClPhTEOS. It is suggested that the layers that the micrographs exhibit are due to the stacking of the less condensed POSS (T_7_ open cages and short ladders), whereas condensed ones (T_8_ close cages) containing only hydrophobic chlorophenyl groups in their T^3^ silicon atoms precipitate and cause the heterogeneity observed in the 20ClPh material. In fact, the aromatic moiety together with the electronic properties of the chlorine atom opens the door to a more specific interaction with volatile aromatic compounds, and thus, for the application of these materials in sensorics for the detection of pollutants in the atmosphere. The study of the chemical and textural properties of these novel hybrid materials is the prelude to their use in different relevant fields. In fact, the aromatic moiety together with the electronic properties of the chlorine atom opens the door to a more specific interaction with volatile aromatic compounds, and thus, to the application of these materials in sensorics for the detection of pollutants in the atmosphere.

## 4. Materials and Methods

### 4.1. Materials

The siliceous precursors TEOS (tetraethoxysilane, purity > 99%) and ClPhTEOS ((4-chlorophenyl) triethoxysilane, purity > 97%) were supplied by Sigma-Aldrich (San Luis, MO, USA). Absolute ethanol (Emsure^®^) and hydrochloric acid (HCl, 37% *w*/*w*) were purchased from Merck (Darmstad, Germany) and potassium bromide (FT-IR grade) from Sigma-Aldrich (San Luis, MO, USA). All chemicals were used as received without further purification.

### 4.2. Synthesis of Silicon Hybrid Xerogels

Hybrid xerogels were prepared following the procedure described in previous works [37,72,73], where the molar ratio of (TEOS + ClPhTEOS): ethanol: water was fixed to 1:4.75:5.5 throughout the whole series, and the amounts of reagent and solvent were adjusted to obtain 20 mL of alcogels. The xerogels were named after the molar percentage used (i.e., 15ClPh for the xerogel with a molar percentage of 15%).

Briefly, TEOS and ClPhTEOS were mixed in a 30 mL vessel (φ 3.5 cm, threaded plastic lid, Schrarlab, Barcelona, Spain). Then, absolute ethanol was added, followed by the dropwise addition of Milli-Q grade water under magnetic stirring to facilitate miscibility. Once the pH of the mixtures remained unchanged (after approximately 10 min), an automatic burette (Tritino mod. 702 SM, Metrohm, Herisau, Switzerland) was used to set the pH at 4.5 (0.05 M HCl), and the mixture was stirred for 10 min to ensure homogenization. The closed vessels were placed in a thermostatized oven at 60 °C (J.P. Selecta S.A, Barcelona, Spain) until gelation (considered the time when the shape of the materials did not change when the vessel was tilted). Subsequently, 5 mL of ethanol was added to cure the alcogel at room temperature for one week. Next, the vessels were opened and covered with parafilmTM, perforated with holes to facilitate solvent evaporation, and dried at room temperature under atmospheric pressure. The monolith was considered dried when no significant variation in its mass was observed. Finally, the xerogels were further dried (90 °C under vacuum) and then ground in an agate mortar.

### 4.3. Characterization of Silicon Hybrid Xerogels

Various techniques have been applied to characterize the structure of the hybrid materials due to their amorphous nature [74]. FT-IR spectra were acquired using a Jasco spectrometer (mod. 4700, Japan). Two spectra were obtained for each sample: (i) In the range of 4000–2200 cm^−1^, using 2 mg of the sample to obtain information on the –OH groups and aromatic C–H bonds, and (ii) in the range of 2200–400 cm^−1^ using 0.6 mg of the sample to avoid saturation of the Si–O–Si asymmetric stretching signal [75]. Tablets were dispersed in KBr and dried overnight. Spectra were recorded using 25 scans and a resolution of 4 cm^−1^. The absorbance was transformed into Kubelka–Munk units with the internal software of the spectrometer (Spectramanager, SMII FTIR Rev 216A ver2.15A) for the deconvolution of the spectra [44]. The method was modified to generate a maximum of fourteen Gaussian–Lorentzian bands in the 1450–700 cm^−1^ spectral range, with a maximum of 200 interactions and a fixed baseline.

^29^Si Cross Polarization Magic-Angle Spinning (CP MAS) solid-state NMR was recorded on a Bruker AV-400 MHz spectrometer (Billerica, MA, USA) operating at 79.5 MHz. Chemical shifts were given in parts per million using TMS as the reference. The spectra were obtained ^1^H decoupled, with a frequency of rotation of 5 kHz and 800 scans per spectrum. For the ^29^Si NMR studies, classical notation was employed: T notation for silicon atoms from the organic precursor (bonded to three oxygens capable of forming siloxane bridges), and Q notation for silicon atoms from TEOS (bonded to four oxygens that can yield siloxane bridges). To describe the number of Si–O–Si bridges in each silicon atom, T and Q notations were completed with a superscript i (T^i^, i = 0, 1, 2 or 3; Q^i^, i = 0, 1, 2, 3 or 4) [62].

X-ray diffractograms were obtained using a PANalytical Empyrean XRD instrument (Empyrean, Almelo, The Netherlands) with a copper rotating anode and a graphite monochromator (at 45 kV and 40 mA) to select the CuK_α1/2_ wavelength at 1.54 nm. Measurements were performed in a stepped scan mode (steps of 0.013°), in the range of 2 ≤ 2θ ≤ 50° at a rate of 0.5 steps s^−1^ [76].

A helium pycnometer was used to measure the skeletal density (AccuPyc 1330, Micromeritics, Norcross, GA, USA), performing 20 purges and 10 measurements for the initial calibration. The sample was weighed into a 1 cm^3^ cell and analyzed using 10 purges and 5 measurements.

N_2_ adsorption isotherms (at −196 °C with an isothermal jacket and immersed in a Dewar with liquid nitrogen) and CO_2_ adsorption isotherms (at 0 °C in a thermostatized recirculation bath using ethylene glycol as a refrigerant) were determined with a volumetric adsorption system (ASAP2020, Micromeritics, Norcross, GA, USA). First, 150 mg of sample was used for each isotherm, placed into a Pyrex glass tube, and degassed at 150 °C up to a residual vacuum of less than 0.66 Pa. The time needed for the analysis ranged from 14 to 55 h for N_2_ adsorption and from 2.5 to 9 h for CO_2_ adsorption. The recorded adsorption data were analyzed with the Microactive software (version 4.06), adjusting the parameters as appropriate for each model. Specific surface areas were calculated using two techniques: (i) The Brunauer–Emmett–Teller (BET) model (a_BET_), applying the Rouquerol criteria [77] and (ii) the Dubinin–Radushkevich (DR) method, applying a molecular section of 0.17 nm^2^ to CO_2_ (a_DR_) [78]. Pore volumes were defined by their diameter (φ): (i) The volume of micropores (φ ≤ 2 nm) was obtained from the DR method (V_micro(N2)_ and V_micro(CO2)_), (ii) the volume of mesopores (2 < φ ≤ 50 nm) was calculated by abstracting the amount of N_2_ adsorbed at p/p° = 0.3 from that adsorbed at p/p° = 0.8 (V_meso(N2)_), and (iii) the total volume (V_Total(N2)_) was considered the amount of N_2_ adsorbed at p/p^o^ = 0.95. Liquid densities of the adsorbates were obtained from the literature (0.808 g cm^−3^ for N_2_ and 1.023 g cm^−3^ for CO_2_) [70,79]. Porosity distributions were determined according to density-functional theory (DFT) using SAIEUS software and applying the “carbon-N2-77, 2D-NLDFT heterogeneous Surface” model for N_2_ adsorption and the “carbon-CO2-273, 2D-NLDFT Het Surface, pp max = 10 atm” model for CO_2_ adsorption. Mean pore sizes were further determined by applying the Barrett–Joyner–Halenda (BJH) method to the desorption curves, using a Kruk–Jaroniec–Sayari correction and thickness curve.

Field-emission scanning electron microscopy (FE-SEM) provides high-resolution images of sample surfaces. Micrographs were obtained with a Carl Zeiss SMT field emission scanning electron microscope (Carl Zeiss SMT, Oberkochen, Germany) at 200 kV. 

High-resolution transmittance electron microscopy (HR-TEM) images were obtained at 200 kV with a JEOL 2000FXII microscope (Jeol Ltd., Tokyo, Japan) with a 0.28 nm point-to-point spatial resolution. The instrument was equipped with an Energy Dispersive X-Ray (EDX) INCA 200 spectrometer (Oxford Instruments, Abingdon, UK), which allowed the observation of the atomic distribution of carbon, silicon, and chlorine on the material.

## Figures and Tables

**Table 1:** List of FT-IR bands of the 100ClPh spectrum and proposed assignation based on the literature.

N°	Wavelength	Vibration	Structural	Reference
	(cm^−1^)	Assignation	Unit	
1	1380	ν (C=C)	Si–Ph–Cl	[36,48]
2	1190	ν_as_ (Si–O–Si), LO mode	(SiO)_6_	[40,44]
3	1160	ν_ring-as_ (Si–O–Si), LO mode	(SiO)_4_; T_7_	[49]
4	1135	ν_ring-as_ (Si–O–Si), LO mode	(SiO)_4_; T_8_	[40,44,49,50,51]
5	1120	ν_ring-s_ (Si–O–Si), LO mode	(SiO)_4_; T_7_, Sld	[49]
6	1085	ν_as_ (C=C)	Si–Ph–Cl	[48]
7	1050	ν_as_ (Si–O–Si), TO mode	(SiO)_4_, Sld	[40,44,49,51]
8	1030	ν_as_ (Si–O–Si), TO mode	(SiO)_6_	[40,44]
9	1015	ν_s_ (C=C)	Si–Ph–Cl	[48]
10	1005	ν_as_ (Si–O–Si)	Lineal siloxane	[50]
11	950	ν (Si-O^−^/Si-OH)	Si–OH	[40,44,46]
12	815	Τ_δ,y_ C–H	Si–Ph–Cl	[36,48]
13	760	Φ C–H	Si–Ph–Cl	[36]
14	710	C–Cl	Si–Ph–Cl	[36,47]

ν, Stretching vibration; ν_s_, Symmetric stretching vibration, ν_as_, Asymmetric stretching vibration; ν_ring-s_, Antiparallel displacements of O atoms on opposite sides of a (SiO)_x_ ring; ν _ring-as_, Parallel displacements of O atoms on opposite sides of a (SiO)_x_ ring; Τ_δ,y_ C-H, Wagging out and inside the plane; Φ, Deformation out and inside the plane; LO, Longitudinal optical vibration mode; TO, Transversal optical vibration mode; (SiO)_4_, 4-fold ring; (SiO)_6_, 6-fold ring; T_7_, Open cage-like silsesquioxane; T_8_, Cage-like silsesquioxane; SLd, Ladder-like silsesquioxane.

**Table 2:** Proportion of (SiO)_4_ and (SiO)_6_ rings in the hybrid materials.

Hybrid Material	LO_6_	LO_4_	TO_4_	TO_6_	(SiO)_4_	(SiO)_6_
	(%)				(%)	
0ClPh	7.0	28.5	18.1	46.5	46.5	53.5
5ClPh	26.7	31.8	38.6	3.0	70.4	29.6
10ClPh	12.8	43.9	29.8	13.6	73.7	26.3
15ClPh	12.3	43.1	33.9	10.7	77.0	23.0
20ClPh (Monolith)	3.3	13.4	30.9	52.4	44.3	55.7
20ClPh (Precipitate)	2.6	21.0	68.3	8.1	89.6	10.8
100ClPh	0.4	77.2	20.2	2.3	97.4	2.7

**Table 3:** Chemical shifts and integral areas of the ^29^Si NMR spectra of the hybrid materials.

Xerogel	^29^Si NMR (ppm)					Band Areas			
	T^2^	T^3^	Q^2^	Q^3^	Q^4^	T	T^2^	T^3^	T^3^/T^2^
0ClPh	^a^	^a^	−92.1	−100.9	−109.0	^a^	^a^	^a^	-
1ClPh	^a^	^a^	−92.3	−101.0	−109.3	^a^	^a^	^a^	-
5ClPh	−69.8	−78.7	−92.3	−101.1	−109.4	5.5	4.7	0.8	0.2
10ClPh	−69.8	−79.1	−92.2	−101.0	−108.5	12.8	8.4	4.4	0.5
15ClPh	−69.8	−78.2	−92.1	−100.9	−108.6	17.6	11.1	6.5	0.6

^a^ Non detected.

**Table 4:** Bragg angles (2θ), band area (A), and bond distance (d_1_ and d_2_ (nm)) calculated from XRD maxima of hybrid xerogels at different molar percentages of organic precursor (ClPhTEOS).

Xerogel	Peak 2θ < 10°			Peak 10° > 2θ < 30°		
	2θ_1_ (°)	A_1_	d_1_ (nm)	2θ_2_ (°)	A_2_	d_2_ (nm)
0ClPh	_a_	_a_	_a_	24.11	6481	0.369
1ClPh	_a_	_a_	_a_	23.93	6845	0.372
5ClPh	_a_	_a_	_a_	23.58	5921	0.377
10ClPh	3.6	2112	2.43	24.14	5778	0.369
15ClPh	4.1	1985	2.17	24.53	5534	0.363

^a^ Non detected.

**Table 5:** Textural parameters of the hybrid xerogels.

Xerogel	a_BET_	a_DR_	V_micro_	V_micro_	V_meso_	V_total_	BJH APS ^a^	E_c_ ^b^	E_c_ ^b^
	(N_2_)	(CO_2_)	(N_2_)	(CO_2_)	(N_2_)	(N_2_)		(N_2_)	(CO_2_)
	(m^2^ g^−1^)		(cm^3^ g^−1^)				(nm)	(KJ mol^−1^)	
0ClPh	697	510	0.283	0.195	0.074	0.407	3.61	15.27	19.71
1ClPh	656	426	0.253	0.163	0.222	0.557	4.38	15.57	19.97
3.5ClPh	504	429	0.205	0.164	0.007	0.223	3.33	18.73	19.28
5ClPh	493	400	0.205	0.153	0.006	0.209	3.40	18.11	19.77
7.5Clph	431	388	0.176	0.148	0.004	0.177	3.29	19.49	18.92
10ClPh	367	363	0.151	0.139	0.003	0.147	2.05	16.24	19.29
15ClPh	497	358	0.208	0.137	0.007	0.212	3.11	15.61	19.55

^a^ Average Pore Size from desorption loop; ^b^ Characteristic energy from Dubinin–Raduskevich.

**Table 6:** Average weight percentage of chlorine determined theoretically and by EDX of the hybrid materials.

Xerogels	Weight Percentage of Chlorine		E/T
	(wt%)		
	Theoretical (T)	Experimental (E)	
1ClPh	0.37	0.29	0.77
5ClPh	1.68	1.20	0.72
10ClPh	2.97	2.80	0.94
15ClPh	3.99	2.28	0.57

## Data Availability

The data presented in this study are available on request from the corresponding author.

## Supplementary Materials

The following supporting information can be downloaded at: https://www.mdpi.com/article/10.3390/gels8100677/s1, Figure S1. FTIR spectra (range 4000–2750 cm^−1^) of the hybrid materials at different molar percentages and the reference (0ClPh); Figure S2. Photographs of the ClPhTEOS materials: (a) 0Clph, (b) 15Clph, (c) 20ClPh, and (d) 100ClPh; Figure S3. Calculated spectra and Gaussian–Laurentzian bands generated in the curve fitting for: (a) 5ClPh, (b) 10ClPh, (c) 15ClPh, (d) 20ClPh (monolith), and (e) 20ClPh (precipitate); Table S1. Wavelength and percentual area of the generated bands in the curve fitting for each ClPhTEOS material, and the curve fitting parameters; Figure S4. XRD pattern of 100ClPh.
