# Supplementary material for: Novel Silica Hybrid Xerogels Prepared by Co-Condensation of TEOS and ClPhTEOS: A Chemical and Morphological Study
Source: Gels. 2022 Oct 20;8(10):677. doi: 10.3390/gels8100677 (PMC9601464; doi:10.3390/gels8100677)
Supplement: Supplementary file 1 [file gels-08-00677-s001.zip › gels-1970971-supplementary.pdf]

SUPPLEMENTARY MATERIAL

Novel Silica Hybrid Xerogels Prepared by Co-Condensation of TEOS and ClPhTEOS: A Chemical and Morphological Study

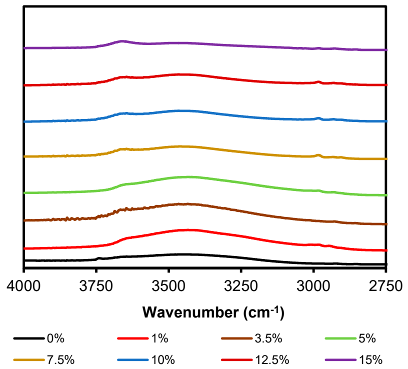

Figure S1. FTIR spectra (range 4000-2750 cm^{-1}) of the hybrid materials at different molar percentages and the reference (100%TEOS).

(a) **(b)** **(c)** **(d)**

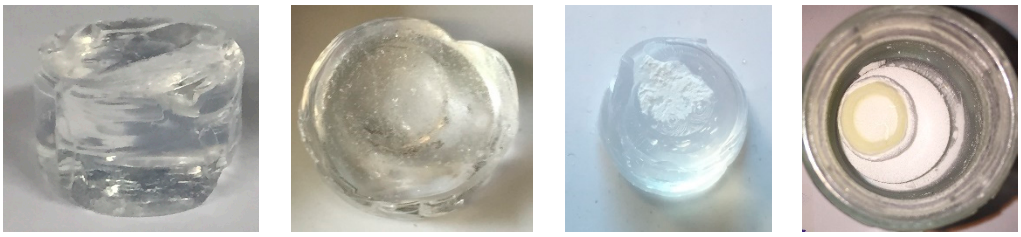

Figure S2. Photographs of ClPhTEOS materials: (a) 0Clph, (b) 15Clph, (c) 20ClPh, and (d) 100ClPh.

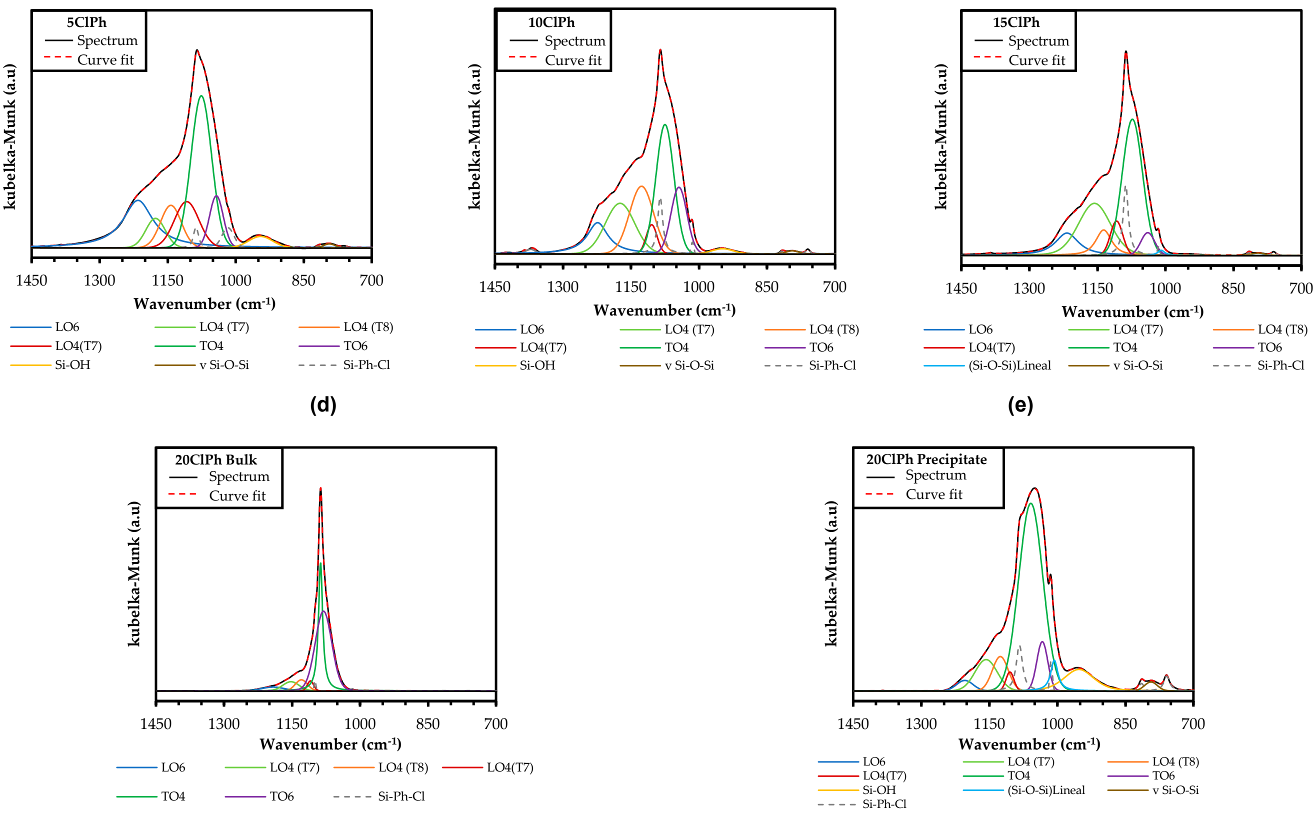

Figure S3. Synthetic spectra and Gaussian-Laurentzian bands generated in the curve fitting for: (a) 5ClPh, (b) 10ClPh, (c) 15ClPh, (d) 20ClPh(monolith) and (e) 20ClPh(Precipitate).

Table S1. Wavelength and porcentual area of the generated band in the curve fitting for each ClPhTEOS material, and the curve fitting parameters.

N° of band	0ClPh		5ClPh		10ClPh		15ClPh		20ClPh (Monolith)		20ClPh (Precipitate)		100ClPh	
	cm^{-1}	Area (%)	cm^{-1}	Area (%)	cm^{-1}	Area (%)	cm^{-1}	Area (%)	cm^{-1}	Area (%)	cm^{-1}	Area (%)	cm^{-1}	Area (%)
1	[a]	-	-	-	1371	0.6	1385	0.1	[a]	-	-	-	1385	0.78
2	1227	6.9	1216	23.3	1223	11.6	1193	14.5	1192	3.3	1204	2.1	1193	0.2
3	1182	19.4	1177	6.1	1174	17.4	1157	34.8	1153	5.9	1157	8.2	1157	32.8
4	1146	6.7	1143	9.5	1127	19.1	1135	6.4	1129	5.1	1126	6.4	1135	7.9
5	1127	2.1	1108	12.1	1104	3.7	1114	9.6	1110	2.4	1105	1.8	1114	2.5
6	[a]	-	1088	1.5	1085	5.1	1086	10.1	1099	0.7	1084	4.9	1086	16.4
7	1094	17.9	1076	33.6	1075	27.3	1050	39.8	1087	30.7	1059	53.3	1050	11.3
8	1077	46.2	1043	7.7	1044	12.5	1033	12.6	1081	52.0	1033	6.3	1033	1.3
9	[a]	-	1017	2.6	1015	0.3	1015	0.4	[a]	-	1014	0.9	1015	2.0
10	[a]	-	[a]	-	1007	0.6	1008	0.7	[a]	-	1006	3.9	1008	17.0
11	943	0.4	945	2.9	948	1.3	[a]	-	[a]	-	953	8.9	[a]	-
12	[a]	-	815	0.1	816	0.1	815	0.2	[a]	-	815	0.5	813	1.7
13	802	0.3	794	0.5	794	0.4	796	0.6	[a]	-	794	1.4	757	5.7
14	[a]	-	761	0.1	760	0.2	762	0.3	[a]	-	759	1.5	708	0.6

Curve fitting parameters

	0ClPh	5ClPh	10ClPh	15ClPh	20ClPh (Monolith)	20ClPh (Precipitate)	100ClPh
N° of Iterations	116	200	200	200	50	200	38
Residual value	0.00520836	0.00137787	0.00148709	0.00167645	0.894758	0.00043146	0.000698583

[a]Non generated during the curve fitting

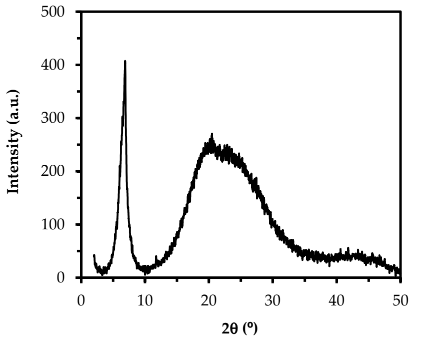

Figure S4. XRD spectrum of 100ClPh
